# A prospective randomized clinical trial to evaluate wound healing, cosmetic and functional results as well as postoperative adverse events comparing two types of dressing technique after hypospadias repair

**DOI:** 10.1007/s00383-025-06063-1

**Published:** 2025-06-18

**Authors:** Kristin Lawo, Dominique Feller, Birgit S. Klein, Stefan G. Holland-Cunz, Martina Frech-Dörfler

**Affiliations:** 1 Department of Pediatric Surgery, University Children’s Hospital Basel, Basel, Switzerland; 2 University of Basel, Basel, Switzerland

**Keywords:** Hypospadias, Postoperative dressing, Children, Urethroplasty, Complications

## Abstract

**Background:**

Dressing technique after hypospadias repair is one of the most controversially discussed subjects in the aftercare. Although current literature presents an enormous number of publications regarding postoperative dressing techniques, there is still no consent on how and if to perform postoperative dressing. The aim of this study is to evaluate two techniques (circular film dressing vs. silver pad covered with film dressing) frequently used in clinical routine after hypospadias surgery.

**Methods:**

Primary outcomes of this prospective randomized clinical trial were wound healing using Southampton Wound Assessment Scale (SWAS) and cosmetic and functional result using Hypospadias Objective Penile Evaluation (HOPE)-Score. Secondary outcomes were postoperative adverse events.

**Results:**

44 patients were included and randomized into two groups (*n* = 24 for film dressing vs. *n* = 20 silver pad dressing). Concerning the primary outcomes, no significant difference in SWAS and HOPE score could be shown. Secondary outcomes showed a statistically significant higher rate of wound dehiscence in the silver pad group and no other statistically significant differences.

**Conclusions:**

This study could not show superiority of one dressing regarding wound healing or functional and cosmetic result and thereby strengthens the assumption that the individual dressing technique does not play a crucial role in the postoperative outcome.

## Introduction

Hypospadias is one of the most common malformations in newborns with an incidence of 0.3–0.7% in male liveborns [1]. The types of malformation vary between distal (glandular and coronal) hypospadias, which are more or less an aesthetic dysfunction, to proximal (penile and penoscrotal) malformation which lead to eminent dysfunction in sexual and urinary function.

While a standardarization in the operative techniques to perform hypospadias repair could be established, the opinions on the best aftercare differ widely. Dressing technique after hypospadias repair is one of the most controversially discussed subjects in the postoperative treatment as it is a burden of care for the parents, reason for frequent outpatient consultations and a financial load with potentially no positive effect [2–5]. Until today, there is no consent on how and if to perform postoperative dressing [6–8].

For years, postoperative dressings after hypospadias repair have been performed with an adhesive circular film dressing (Tegaderm™) at our institution. However, a newer trend using a non-adhesive silver dressing (Polymem Silver®) covering the ventral suture, which is covered with a circular film dressing has emerged in the last years. This study aims to compare the two dressing techniques used in our clinical routine regarding wound healing, functional and aesthetic results as well as postoperative adverse events to decide which dressing is associated with a better outcome so the choice of postoperative dressing is evidence-based and not solely based on surgeons’ preference.

## Materials and methods

**Fig. 1:**
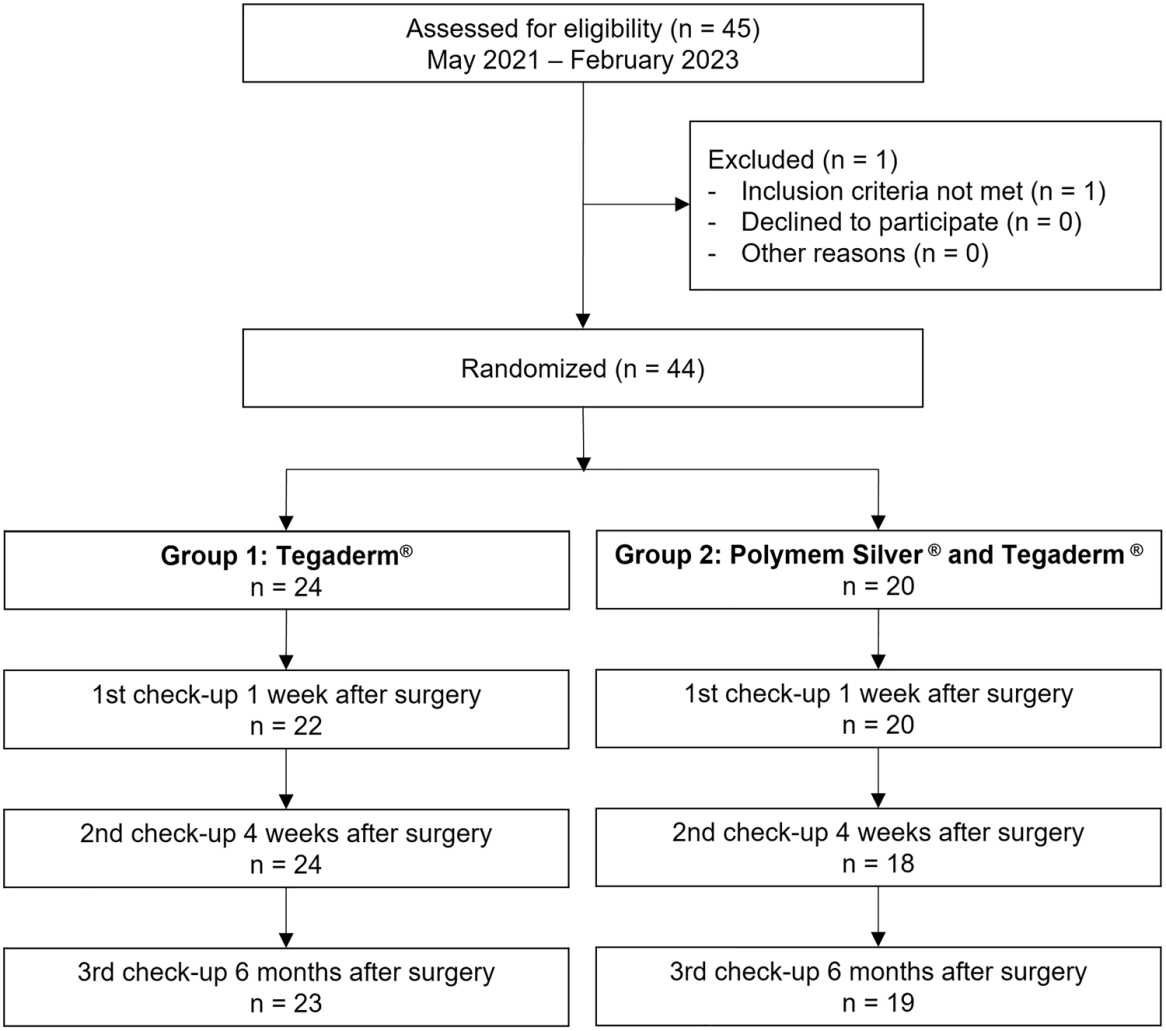
Study design

Approval for this prospective randomized single-centre clinical trial was obtained by the local ethics committee (2020–00948). The inclusion period was from May 2021 to February 2023. Inclusion criteria were an age between 0 and 18 years, presence of hypospadias malformation and written consent of the parents. Exclusion criteria were redo surgeries. Randomization was preoperatively performed by chance into two groups. Surgery technique was chosen according to the severity grade of hypospadias and a transurethral stent was inserted in all patients with urethral reconstruction. No patient fulfilled the indication for additional insertion of a suprapubic catheter. Surgery was performed by 4 different surgeons, while one of the surgeons was present in all cases. Postoperatively, patients in group 1 received an adhesive circular film dressing (Tegaderm™) of the whole penis from the basis to the tip of the glans. In group 2, a non-adhesive silver pad (Polymem Silver®) was cut fitting exactly over the ventral suture of the penile shaft and then was adapted with a circular film dressing (Tegaderm™). In both groups double diaper technique was used. The dressing remained in place until days 7–10 after surgery unless it fell off on its own or there were signs of wound infection. If the dressing did not fall off on its own, the parents were instructed to remove it after soaking in water at home on the day of the first outpatient follow-up. After discharge, three outpatient follow-ups were scheduled 1 week, 4 weeks and 6 months after surgery (see Fig. 1).

Primary outcomes were wound healing assessed using Southampton Wound Assessment Scale (SWAS) and cosmetic and functional result assessed using Hypospadias Objective Penile Evaluation (HOPE)-Score (see Table 1 and 2) [9, 10]. Secondary outcomes were postoperative adverse events, e.g., dislodgement of catheter or dressing, wound healing and urethroplasty associated complications. The evaluation of HOPE-Score and SWAS was done by a paediatric surgery resident who had at least completed 3 years of training in paediatric surgery using photographs taken during the routine follow-up visits in the outpatient department. Data analysis was done single-blinded regarding the dressing technique. Patients were excluded if more than one follow-up was missed.

**Table 1:** Hypospadias Objective Penile Evaluation-Score [9]

1. Position meatus
Orthotopic (10 points)
Coronal (8 points)
Subcoronal (5 points)
Distal shaft (3 points)
Proximal shaft (1 point)
2. Shape meatus
Normal (10 points)
Slightly abnormal (7 points)
Moderately abnormal (4 points)
Severely abnormal (1 point)
3. Shape glans
Normal (10 points)
Slightly abnormal (7 points)
Moderately abnormal (4 points)
Severely abnormal (1 point)
4. Shape skin
Normal (10 points)
Slightly abnormal (7 points)
Moderately abnormal (4 points)
Severely abnormal (1 point)
5.1 Torsion
0–30° (10 points)
30–50° (7 points)
50–70° (4 points)
> 70° (1 point)
5.2 Curvature in penile erection
No erection observed (5.2 does not account for the HOPE-Score)
0–30° (10 points)
30–50° (7 points)
50–70° (4 points)
> 70° (1 point)
Hypospadias Objective Penile Evaluation (HOPE)-score = mean number of points question 1–5

**Table 2:** Southampton Wound Assessment Scale [4]

Grad		Appearance
0		Normal healing
1		Normal healing with
a		Some bruising
b		Considerable bruising
c		Mild erythema
2		Erythema plus other sings of inflammation
a		At one point
b		Around sutures
c		Along wound
d		Around wound
3		Clear or hemoserous discharge
a		At one point only (< 2 cm)
b		Along wound (> 2 cm)
c		Large volume
d		Prolonged (> 3 days)
Major complication		
4		Pus
a		At one point only (< 2 cm)
b		Along wound (> 2 cm)
5		Deep or severe wound infection with or without tissue breakdown, hematoma requiring aspiration

### Statistics

Statistical analysis was performed using IBM® SPSS® Statistics (Version 27.0.0.0). Descriptive data analysis was performed using n (%) for discrete variables and mean ± standard deviation (SD) for continuous variables. To compare the study groups, Mann–Whitney *U* test was performed as normal distribution was not given. All statistical decisions were made on a two-tailed basis with a significance level of *p* < 0.05.

## Results

44 patients meeting the inclusion criteria underwent hypospadias repair. No patient missed more than one follow-up appointment, so no patient was excluded. Localization of hypospadias was distal (glandular, coronal, distal shaft) in 32 patients (72.7%) and proximal (mid shaft, proximal shaft, penoscrotal) in 12 patients (27.3%) without statistically significant difference between groups. A circular film dressing was performed in 24 patients (54.5%; group 1), a silver pad dressing covered with a circular film in 20 patients (45.5%; group 2). There was no significant difference in demographic data, type of hypospadias, surgical technique, usage of suture material and gauge and timing of follow-up between the two groups (see Table 3). A subgroup analysis regarding performing surgeons has not been performed due to lack of power because of the small sample size.

**Table 3:** Demographic data

Patient demographics	Group 1: Adherent film dressing (*n* = 24)	Group 2: Non-adherent silver pad covered with film dressing (*n* = 20)	*p* value
Age at surgery (SD), months	14.5 (14.7)	16.6 (11.9)	0.151
Type of hypospadias			
Distal	18 (75)	14 (70)	1.0
Proximal	6 (25)	6 (30)	1.0
Surgery technique	15 (62.5)	7 (35)	n/a
TIP (%)	1 (4.2)	3 (15)	
Duckett (%)	2 (8.3)	6 (30)	
MAGPI (%)	6 (25)	4 (20)	
Others (%)	15 (62.5)	7 (35)	
Duration of surgery (SD), minutes	103.0 (39.5)	116.4 (47.1)	0.443
Insertion of urethral stent (%) (Charrière 8 Firlit Kluge stent)	18 (75)	15 (75)	1.0

**Fig. 2:**
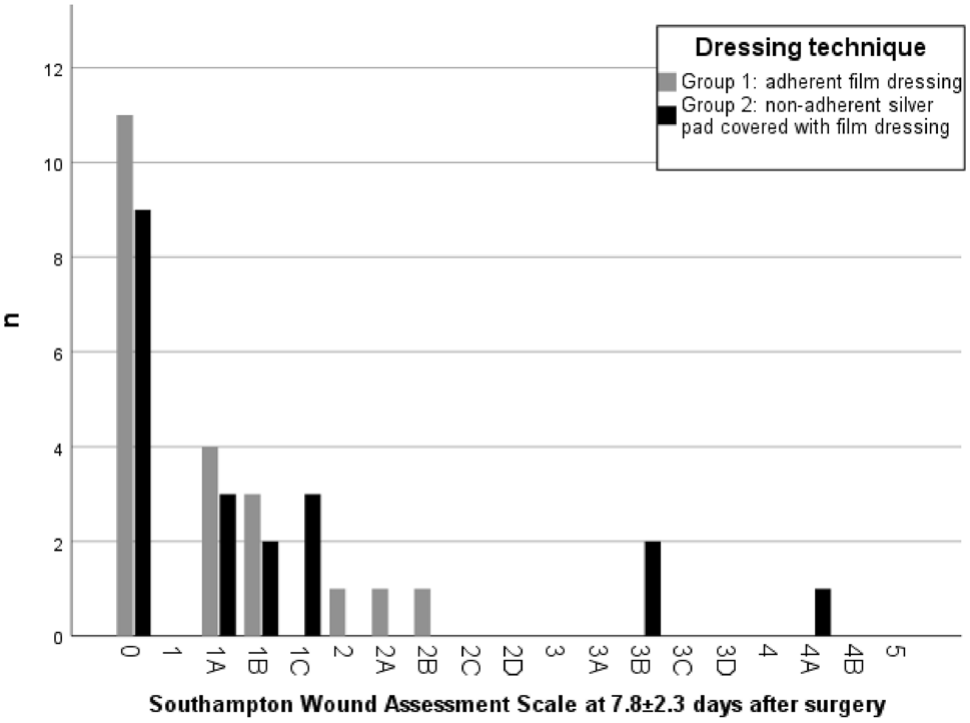
Results of Southampton Wound Assessment Scale

Regarding the primary outcomes, SWAS was performed after a mean of 7.84 ± 2.32 days after surgery. No statistically significant difference of SWAS results could be detected between groups (*p* = 0.427), neither could we detect a significant difference in a sub-analysis focusing on mild (SWAS 0–3) vs. severe (SWAS 4–5) wound healing complications (*p* = 0.542). There was only one patient in the study group with SWAS 4A and need for antibiotic treatment in group 2 treated with a silver pad dressing (see Fig. 2).

HOPE Score was performed after a mean of 29.91 ± 11.7 weeks after surgery and was 9.6 ± 0.67 in group 1 and 9.5 ± 0.66 in group 2 without statistically significant difference (*p* = 0.724).

Mean length of stay of the dressing was 5.29 ± 2.28 days in group 1 vs. 3.94 ± 2.27 days in group 2, mean length of stay of the transurethral catheter was 6.82 ± 2.87 vs. 6.40 ± 4.29. No statistically significant difference was detected in both analyses (*p* = 0.083 and *p* = 0.783).

Secondary outcomes were adverse events after surgery including postoperative bleeding and hematoma, surgical site and urethroplasty associated complications and dislodgement of catheter or dressing. Regarding early in-hospital complications, accidental dislodgement of dressing was statistically significant more frequent in the group treated with the silver pad dressing with 7 cases (35%) vs. 2 cases (8.3%) in the group treated with the film dressing (*p* = 0.031). At the first outpatient follow-up after discharge after a mean of 7.84 ± 2.32 days after surgery, the rate of wound dehiscence was statistically significant more frequent in the group 2 treated the silver pad dressing (*n* = 6 (39%) vs. *n* = 1 (4.2%), *p* = 0.029). One patient in group 2 presented with wound infection with need for antibiotic treatment.

At the second outpatient follow-up after a mean of 28.44 ± 10.69 days after surgery no statistically significant differences were detected. The last follow-up was performed after a mean of 29.91 ± 11.7 weeks after surgery without statistically significant difference. In group 1 treated with the film dressing, 5 patients (20.8%) presented with uretherocutaneous fistula vs. 3 patients (15%) in group 2 without statistically significant difference (*p* = 0.629).

## Discussion

Dressing technique after hypospadias repair is one of the most controversially discussed subjects in the aftercare. Current literature presents an enormous number of publications regarding postoperative dressing techniques including adherent, non-adherent, and glue-based dressings [2, 7, 11–13]. However, no consent on the ideal postoperative dressing has been achieved [6, 7]. Dressings after hypospadias repair are applied to prevent surgical site infections by reducing bacterial contamination and soiling and thereby support wound healing. Furthermore, these dressings help to prevent postoperative bleeding and hematoma and hold the penis in an straight position [14].

Having used an adherent film dressing after hypospadias repair for years at our clinic, we started to apply this in combination with a non-adhesive silver pad, as the removal from the wound is meant to be less traumatic and we were treating burn injuries effectively with this type of dressing seeing almost no infections.

The use of both techniques in our randomized trial was clinically safe as no allergic reaction or skin irritation occurred. A significant difference between patients treated with the silver pad dressing compared to the film dressing could be detected regarding wound dehiscence at the first outpatient follow-up with higher incidence in the latter. One patient in the silver pad group presented with wound infection with need for antibiotic treatment at the first check-up.

As Polymem Silver®, the used non-adhesive silver pad, is an anti-inflammatory, polymeric membrane dressing containing surfactant, glycerin and elemental silver, we hypothesized it to be a good non-adhesive dressing to support wound healing [15]. Our data showed that these characteristics did not prevent more wound healing complications compared to a film dressing alone. The film dressing tended to last longer, possibly because of its adhesiveness. The higher accidental loss of dressing in group 2 treated with the silver pad could be the reason of higher rates of wound dehiscence and postoperative surgical site infections. In addition, the waterproof film dressing may have led to lower wound healing complications by protecting the wound from contamination and thereby providing a supportive wound healing environment by pooling of wound exudates containing proteins, enzymes, and phagocytic cells [16, 17].

However, while the film dressing alone was superior to the silver pad dressing in preventing early postoperative wound dehiscence, overall SWAS results were good in both groups. This indicates that wound healing in general was independent from dressing technique. Regarding postoperative results 6 months after surgery, group 2 treated with the silver pad showed a lower rate of urethrocutaneous fistula, while no statistically significant difference could be found, probably because of the small sample size. As the film dressing is an adhesive, semi-occlusive dressing, it is meant to fall off on its own. We instructed the parents to remove the dressing after soaking in water. Probably this was not sufficient to loosen the dressing and wound microtrauma occurred during removal leading to skin deficiency and fistula. As Sanders et al. showed, the use of an adhesive dressing remover (e.g., Cavilon®, Hexamethyldisiloxan) reduces pain associated with dressing removal and removal time. This could be a good addition to remove postoperative adherent dressings more easily [18].

Occurrence of urethrocutaneous fistula and meatal stenosis might also be influenced by the usage of transurethral stenting and additional insertion of a suprapubic catheter [19]. While this has not been studied in the present clinical trial it may be a confounder on the postoperative outcome and should be investigated by further research.

The lacking statistical difference regarding the HOPE-Score and the rate of uretherocutaneous fistula between the groups at the 6 month follow-up hints to the fact, that the surgeons experience and technique as well as the individual wound healing is more determinizing to the functional and aesthetic result than dressing technique.

Our data are in line with current literature, especially with the review of Escolino et al. published in 2023, who could not prove a beneficial effect of postoperative dressings after hypospadias correction surgery [6]. This suggests not to perform any dressing in the future. However, it must be taken into consideration that two of the analyzed papers in this review are more than 20 years and study design and dressing technique is very heterogenic [6, 8, 20, 21]. Furthermore, a publication bias is highly probable in those studies.

Large, multi-center randomized controlled trials are needed to evaluate the benefits of postoperative dressings after hypospadias correction surgery.

This study has several limitations. The biggest limitation is its small study population, limiting its power. In addition, surgery was performed by four different surgeons which may be a confounder in the analysis.

## Conclusion

In line with current literature this study could not show superiority of one dressing technique neither regarding wound healing nor functional and cosmetic result and thereby strengthens the assumption that the individual dressing technique does not play a crucial role in the postoperative outcome. Further investigation in larger prospective randomized trials is needed.

## Data Availability

No datasets were generated or analysed during the current study.
